# Neutrophil Gelatinase Associated Lipocalin (NGAL) – a biomarker of renal dysfunction in patients with liver cirrhosis: Do we have enough proof?

**Published:** 2015

**Authors:** SG Firu, CT Streba, D Firu, DE Tache, I Rogoveanu

**Affiliations:** * Department of Medical Sciences I, University of Medicine and Pharmacy Craiova, Romania; ** Department of Medical Sciences II, University of Medicine and Pharmacy Craiova, Romania; *** Department of Biochemistry, University of Medicine and Pharmacy Craiova, Romania

**Keywords:** Neutrophil Gelatinase-Associated Lipocalin (NGAL), kidney biomarker, renal dysfunction, liver cirrhosis

## Abstract

**Rationale.** Renal dysfunction has a serious impact on the natural evolution of liver cirrhosis. Treatment and prognosis may be improved if an early diagnosis could be established, and specific therapeutic interventions would be applied. Although RIFLE and AKIN classifications have been successfully implemented in the clinical practice of Nephrology and Intensive Care Units, these did not provide major improvements in patients with liver cirrhosis. In the last decade, various biomarkers of kidney injury have been assessed, and Neutrophil Gelatinase-Associated Lipocalin (NGAL) is one of the most promising and most studied novel biomarker.

**Objective.** To offer a brief evaluation on current data on the utility of this biomarker in patients with liver cirrhosis.

**Methods and results.** We have searched through current literature and analyzed all significant full text articles on this topic.

**Discussions.** NGAL and other new kidney injury molecules may be useful in patients with liver cirrhosis, particularly in identifying structural kidney dysfunction, but larger validation studies to confirm this observation are needed.

**Abbreviations:** ADQI = Acute Dialysis Quality Initiative, AKI = acute kidney injury, AKIN = Acute Kidney Injury Network, ATN = acute tubular necrosis, CKD = chronic kidney disease, Cys C = cystatin C, GFR = glomerular filtration rate, HRS = hepatorenal syndrome, IAC = International Ascites Club, IL-18 = interleukin-18, KIM-1 = kidney injury molecule-1, L-FABP = liver-type fatty acid-binding protein, LT = liver transplantation, MDRD6 = Modification of Diet in Renal Disease 6, NAG = N-acetyl-β-D-glucosaminidase, NGAL = Neutrophil Gelatinase-Associated Lipocalin, pi-GST = pi-glutathione S-transferase, PRA = prerenal azotemia, RBP = retinol binding protein, RRT = renal replacement therapies, SCr = serum creatinine, SLKT = simultaneous liver and kidney transplant, UO = urine output, γ-GT = γ-glutamyl transpeptidase

## Introduction

Kidney dysfunction is a complex and common event in patients with liver cirrhosis. Although novel treatments have shown some promising results [**1**], acute kidney injury (AKI) remains a major complication of decompensated liver cirrhosis with high morbidity and mortality rates [**2**,**3**]. AKI occurs in up to 19-20% of hospitalized patients with liver cirrhosis and among the most frequent causes are prerenal azotemia (PRA), hepatorenal syndrome (HRS), and acute tubular necrosis (ATN), with prevalence rates estimated around 68%, 25%, and 33%, respectively [**2**,**3**]. Reports have shown that approximately 1% of cirrhotic patients with azotemia suffer from progressive parenchymal renal disease secondary to hepatic viral infections, immune or metabolic disorders (chronic glomerulonephritis, IgA nephropathy, diabetic nephropathy) [**2**,**4**].

## Definitions of AKI and CKD

In 2011, after a joint debate, members of the Acute Dialysis Quality Initiative (ADQI) and the International Ascites Club (IAC) developed a new collection of diagnostic criteria for an improved evaluation of kidney impairment in liver cirrhosis [**5**,**6**]. The term “Acute Kidney Injury (AKI)” is used to describe the abrupt decline of the renal function indicated by a boost in serum creatinine level of >50% from baseline, or by an upward trend in serum creatinine level of ≥26.4 µmol/ L (≥0.3 mg/ dL) in less than 48 hours. Chronic Kidney Disease (CKD) can be defined by an estimated glomerular filtration rate (eGFR) below 60 ml/ minute for more than 3 months, by using the Modification of Diet in Renal Disease 6 (MDRD6) formula (this is considered the most accurate creatinine-based formula in cirrhotic patients). Acute chronic kidney disease manifests as an overlapping of AKI on pre-existing chronic renal disease according to the previous definitions for AKI and CKD [**5**,**6**].

## RIFLE classification

Gathered together in Vicenza (Italy) in May 2002, the members of ADQI group elaborated a new set of diagnostic and classification criteria for AKI: the RIFLE classification (published in May 2004). This classification system includes three classes for severity (Risk, Injury, Failure) and another two classes for outcome (Loss of kidney function, End-stage kidney disease) defined by perturbations in serum creatinine, glomerular filtration rate or urine output as described in **Table 1** [**7**].

**Table 1:** RIFLE classification by ADQI (adapted from references [**5**,**7**,**8**])

Class	GFR criteria	UO criteria	
Risk	↑ SCr × 1.5 or ↓ GFR >25%	<0.5 mL/kg/h × 6 h	Severity classes
Injury	↑ SCr × 2 or ↓ GFR >50%	<0.5 mL/kg/h × 12 h	
Failure	↑ SCr × 3 or ↓ GFR >75% or if baseline SCr ≥353.6 μmol/ L (≥4 mg/ dL) ↑ SCr >44.2 μmol/ L >0.5 mg/ dL)	<0.3 mL/kg/h × 24 h or anuria × 12 h	
Loss of kidney function	Complete loss of kidney function >4 weeks		Outcome classes
End-stage kidney disease	Complete loss of kidney function >3 months		
GFR = glomerular filtration rate; UO = urine output; SCr = serum creatinine			

**AKIN classification**

After a meeting in Amsterdam (September 2005), the Acute Kidney Injury Network (AKIN) group developed a new set of criteria for AKI known as the AKIN classification (published in March 2007). This improved the classification system which consisted of 3 stages of severity and was based only on changes in serum creatinine (2 measurements within 48 h) and urine output as shown in **Table 2** [**7**].

**Table 2:** AKIN classification (adapted from references [**5**,**7**])

Stage	SCr criteria	UO criteria
1	↑ SCr ≥26.5 μmol/ L (≥0.3 mg/ dL) or ↑SCr ≥150-200% (1.5-2×)	<0.5 mL/kg/h (>6 h)
2	↑ SCr >200-300% (>2-3×)	<0.5 mL/kg/h (>12 h)
3	↑ SCr >300% (>3×) or if baseline SCr ≥353.6 μmol/ L (≥4 mg/ dL) an ↑SCr ≥44.2 μmol/ L (≥0.5 mg/ dL)	<0.3 mL/kg/h (>24 h) or anuria (>12 h)
SCr = serum creatinine; UO = urine output. * patients requiring RRT are included independent of the stage		

A common limitation of both classifications systems is their inability to provide any information on the cause of the renal dysfunction in liver cirrhosis. Existing data cannot support the superiority of AKIN classification to traditional criteria regarding risk prediction in patients with liver cirrhosis and renal failure [**9**]. Fagundes et al. observed that a combination between AKIN classification and traditional criteria for kidney impairment might provide a better assessment of risk in patients with liver cirrhosis, compared with AKIN criteria alone [**10**].

Despite the fact that it lacks in accuracy, particularly in the setting of liver cirrhosis, serum creatinine is our current marker for kidney dysfunction, and it is widely used. In patients with liver cirrhosis and CKD, decreased hepatic synthesis of creatinine and significant loss of muscle mass [**3**,**5**] tend to lead to an overestimation of GFR by using creatinine-based formulas. In the setting of AKI, after a renal insult takes place, a steady state must be reached in order to accurately estimate the GFR by using serum creatinine. Most commonly, serum creatinine levels are assessed by using Jaffe reaction, which may interfere with bilirubin levels [**11**] therefore, the enzymatic method for the determination of creatinine would be more appropriate in these scenarios. Besides these shortcomings, serum creatinine does not have the ability to differentiate among the causes of renal impairment [**4**]. 

## NGAL and other markers of kidney injury

The distinction among the main forms of renal impairment is a crucial step because the outcomes and the management are very different. This challenge has led to a continuous research in this area and to the discovery of new markers of kidney dysfunction. These novel biomarkers are usually small molecules (e.g. proteins or enzymes), that are released into the systemic circulation or urine as a result of changes in glomerular filtration rate, tubular cell injury or inflammatory cell infiltration [**12**]. Modern techniques using genomics and proteomics have identified several candidates for the role of kidney biomarkers such as: neutrophil gelatinase-associated lipocalin (NGAL), kidney injury molecule-1 (KIM-1), interleukin-18 (IL-18), liver-type fatty acid-binding protein (L-FABP), cystatin C (Cys C), N-acetyl-β-D-glucosaminidase (NAG), α1/ β2-microglobulin, γ-glutamyl transpeptidase (γ-GT), retinol binding protein (RBP), pi-glutathione S-transferase (pi-GST), etc. [**12**].

Neutrophil gelatinase-associated lipocalin (NGAL), also described as lipocalin-2 or human neutrophil lipocalin, is one of the most commonly studied novel biomarkers. By its affiliation to the lipocalin superfamily, this small glycoprotein has a specific shape that provides an ability to bind and transport different molecules [**13**] and can be found in the circulation as three different structural isomers: a monomer (25-kDa), a homodimer (45-kDa), a heterodimer (135-kDa) [**14**]. NGAL was first discovered inside the specific granules of neutrophils, but further studies revealed that it is secreted by various cells of the body: epithelial cells (lungs, bowel, prostate, kidney, etc.), neutrophils, monocytes/ macrophages, adipocytes [**15**]. There is a low baseline production of NGAL that maintains its serum concentration to around 20 ng/ mL, but various stimuli that induce epithelial damage can increase this baseline level [**14**]. Due to these factors, among the scenarios with an elevated production of NGAL are sepsis, malignancy, chronic kidney disease, pancreatitis, chronic obstructive pulmonary disease, endometrial hyperplasia, urinary tract infection [**12**]. Increased levels of NGAL can be identified in plasma and urine starting 2-4 hours after a kidney injury, resulting from alterations in glomerular filtration and tubular reabsorption and also by increased secretion in tubular epithelial cells [**12**]. Serum NGAL levels seem to be directly correlated with urine NGAL levels, therefore, a urine NGAL/ serum NGAL ratio may prove to be a better marker of renal impairment [**23**].

The utility of NGAL as a biomarker of renal impairment has been tested in various clinical situations, in both adult and pediatric populations: critically ill patients [**3**], after cardiac surgery [**16**-**19**], after contrast agent administration [**20**], sepsis [**21**], trauma patients [**22**].

## Objective (aim)

The purpose of our review is to present a synthesis of the current data regarding the potential applicability of NGAL in daily practice in patients with liver cirrhosis and renal dysfunction. In order to have a better interpretation, we have divided the existing data on this topic, according to different applications of this biomarker in patients with liver cirrhosis. All the studies showed higher levels of NGAL and other markers studied in patients with liver cirrhosis and renal impairment compared with patients without renal dysfunction [**23**-**29**]. 

## NGAL and risk assessment

In terms of risk assessment, Slack and his colleagues [**23**] conducted a small study investigating various methods of assessing glomerular filtration rate and the predictive values of proteinuria and kidney injury biomarkers in AKI development. From a small cohort of 34 patients with liver cirrhosis, eighteen (53%) were diagnosed with AKI. Serum NGAL concentration and Cystatin C-based formulas for the estimation of GFR were good early predictors of AKI, with AUROC values of 0.74 (0.51–0.97), P = 0.04 and 0.72 (0.52–0.92), P = 0.02 respectively. 

## NGAL and differential diagnosis

Verna et al. [**24**] were among the first to study the role of urinary NGAL in patients with liver cirrhosis and renal impairment. One of the results of their study was the ability of uNGAL to discriminate between different types of AKI. By using only one uNGAL measurement at hospital admission, the group of researchers was able to divide patients with AKI into three categories: prerenal azotemia (low levels), hepatorenal syndrome (intermediate levels) and intrinsic AKI (high levels).

A cross-sectional study conducted by Qasem and his colleagues [**25**] evaluated the utility of two urinary biomarkers of kidney damage (Neutrophil Gelatinase-Associated Lipocalin and Interleukin-18). A total of 160 hospitalized patients with liver cirrhosis were divided into three cohorts: non-ascitic patients (n = 42), ascitic patients without renal impairment (n = 50), and ascitic patients with renal impairment (n = 68). Levels of both urinary NGAL and urinary IL-18 were significantly higher in patients with ascites and renal impairment compared to the other two groups. Also, both urinary biomarkers were able to differentiate between causes of AKI, with highest levels in ATN (uNGAL: 580.51±238.75 μg/ g creatinine, uIL-18: 1687±447 μg/ g creatinine), intermediate levels for HRS (uNGAL: 380.6±132.32, uIL-18: 953±273), and the lowest levels in prerenal azotemia (uNGAL: 161.15±60.75, uIL-18: 451.47 ± 121.73). In patients with liver cirrhosis and CKD, the researchers reported medium values of uNGAL (232.63±41.31 μg/ g creatinine) and uIL-18 (582±98.24 μg/ g creatinine), ranged between those of prerenal azotemia and HRS groups.

In a multicenter study, Belcher et al. [**26**] evaluated the utility of different biomarkers of kidney damage as differential diagnosis tools for AKI in 188 cirrhotic patients. The subjects were separated into three major groups depending on the form of AKI: prerenal azotemia (PRA), hepatorenal syndrome (HRS), acute tubular necrosis (ATN). A set of new and traditional urinary biomarkers was assessed: neutrophil gelatinase-associated lipocalin, interleukin-18, kidney injury molecule-1, liver-type fatty acid binding protein, fractional excretion of sodium and albumin. Urinary markers of kidney injury were substantially elevated in patients with liver cirrhosis and AKI induced by ATN.

Fagundes and colleagues [**27**] evaluated the value of urinary NGAL levels in the differential diagnosis of renal impairment in a cohort of 241 patients with liver cirrhosis. Only 84 patients had a grade of impaired kidney function, with higher urinary NGAL levels than the rest of the cohort (patients with and without ascites). Patients with ATN had the most elevated levels of urinary NGAL compared to those of patients with other etiologies of AKI, chronic kidney disease (CKD), and HRS (P < 0.001). For a better interpretation of the results, patients with HRS were divided in three categories (HRS-associated with infections, type-1 HRS and type-2 HRS) with the highest values of uNGAL in the first two groups (median and IQ range 391(72–523) vs. 147(83–263) vs. 43(31–74) μg/ g creatinine). Despite significantly raised levels found in patients with renal impairment, plasma NGAL was not useful in distinguishing between the causes of kidney dysfunction in patients with liver cirrhosis. 

Gungor et al. [**28**] studied the differences in plasma and urine NGAL levels in 64 cirrhotic patients and 23 control subjects. Results of the study showed the highest values of plasma NGAL in patients with type 1 HRS, with statistically significant differences compared to patients with type 2 HRS and compensated liver cirrhosis. Urinary NGAL levels showed the same trend, with greater values in type 1 HRS, intermediate values in type 2 HRS, and low levels in patients with stable liver cirrhosis and controls, without significant differences between type 1 HRS and type 2 HRS patients. Patients with stable liver cirrhosis showed approximately the same values of plasma and urinary NGAL as the control subjects.

## NGAL and prognosis

In their study, Gungor and his colleagues [**28**] revealed a higher mortality rate in patients with HRS, correlated with plasma NGAL levels (deceased patients: 660.4±354.1 μg/ L, surviving patients: 274±289.5 μg/ L, P < 0.001) and urine NGAL levels (deceased patients: 449.6±444.2 μg/ L, surviving patients: 137.2±249.5 μg/ L, P = 0.009).

Baretto et al. [**29**] evaluated the clinical importance of urinary NGAL in the setting of liver cirrhosis and infections. Their study included 132 hospitalized patients with liver cirrhosis and infections and three measurements for uNGAL were carried out at the time of infection diagnosis, on day 3 and day 7. Markedly elevated levels of uNGAL were found among 65 patients clinically diagnosed with AKI, compared to non-AKI patients (203±390 vs. 79±126 μg/ g creatinine, p<0.001), and an even more significant difference was observed between persistent AKI group (n = 4) and transient AKI group (n = 25) (281±477 vs. 85±79 μg/ g creatinine, p<0.001). Patients diagnosed with type-1 HRS showed lower values of uNGAL, therefore this urinary biomarker may have the ability to distinguish type-1 HRS from other causes of persistent AKI (59±46 vs. 429±572 μg/ g creatinine, respectively; p<0.001). High uNGAL levels measured when infection diagnosis was established, were good predictors for the development of a second infection during hospitalization and for the 3-month mortality rate. 

## Discussions

The last decade accounted as the first steps in the new era of kidney damage biomarkers, but after a burst of enthusiasm, clinical studies seemed to bring us down to earth. Although at first neutrophil gelatinase-associated lipocalin (NGAL) was considered a promising new biomarker of renal impairment, current data revealed some limitations of its applicability in clinical practice. It is clear that NGAL can diagnose AKI, but larger studies should clarify the exact cut-off values and the best moment for determination.

These novel biomarkers could be of assistance in the pre- and post-transplant evaluation of patients with liver cirrhosis. Some studies indicated that NGAL and other new biomarkers are good prediction tools for early post-transplant AKI and tacrolimus-induced AKI in patients with liver transplantation (LT) [**30**-**32**]. In the pre-transplant setting, kidney damage biomarkers could recognize patients with structural renal impairment in need of simultaneous liver and kidney transplant (SLKT). 

Although studies have shown that there could be a cut-off value for NGAL that could differentiate patients with HRS, future research is required to identify other specific biomarkers for HRS.

A “modified” AKIN classification may now be the best solution [**10**], but in the future, a classification that associates the AKIN criteria and kidney injury biomarkers levels can be very useful because the cause of kidney dysfunction plays an important role in the prognosis cirrhotic patients.

By including these biomarkers in the clinical management of AKI, we could identify more accurately patients with underlying structural kidney damage and apply the appropriate treatment.

NGAL and other new kidney injury molecules may be useful in patients with liver cirrhosis, particularly in identifying structural kidney dysfunction, but larger validation studies are still needed to confirm this observation. 

**Acknowledgements**

This paper was published under the frame of European Social Found, Human Resources Development Operational Programme 2007-2013, project no. POSDRU/159/1.5/S/133377.

All authors have made substantial and equal contributions to this manuscript.
